# The Mechanisms of Nuclear Proteotoxicity in Polyglutamine Spinocerebellar Ataxias

**DOI:** 10.3389/fnins.2020.00489

**Published:** 2020-06-04

**Authors:** Davin Lee, Yun-Il Lee, Young-Sam Lee, Sung Bae Lee

**Affiliations:** ^1^Department of Brain and Cognitive Sciences, Daegu Gyeongbuk Institute of Science and Technology (DGIST), Daegu, South Korea; ^2^Well Aging Research Center, Division of Biotechnology, Daegu Gyeongbuk Institute of Science and Technology, Daegu, South Korea; ^3^Department of New Biology, Daegu Gyeongbuk Institute of Science and Technology, Daegu, South Korea

**Keywords:** polyQ SCAs, CAG repeat expansion, repeat instability, nuclear translocation, nuclear proteotoxicity

## Abstract

Polyglutamine (polyQ) spinocerebellar ataxias (SCAs) are the most prevalent subset of SCAs and share the aberrant expansion of Q-encoding CAG repeats within the coding sequences of disease-responsible genes as their common genetic cause. These polyQ SCAs (SCA1, SCA2, SCA3, SCA6, SCA7, and SCA17) are inherited neurodegenerative diseases characterized by the progressive atrophy of the cerebellum and connected regions of the nervous system, which leads to loss of fine muscle movement coordination. Upon the expansion of polyQ repeats, the mutated proteins typically accumulate disproportionately in the neuronal nucleus, where they sequester various target molecules, including transcription factors and other nuclear proteins. However, it is not yet clearly understood how CAG repeat expansion takes place or how expanded polyQ proteins accumulate in the nucleus. In this article, we review the current knowledge on the molecular and cellular bases of nuclear proteotoxicity of polyQ proteins in SCAs and present our perspectives on the remaining issues surrounding these diseases.

## Introduction

Spinocerebellar ataxias (SCAs) are neurodegenerative diseases that cause progressive loss of cerebellar neurons and connected regions of the nervous system, resulting in unsteady gait, clumsiness, and dysarthria (Buijsen et al., 2019). With over 32 subtypes linked to differing age of onset, toxic phenotype, and origin of disease, SCAs are neurodegenerative diseases that are clinically and genetically heterogenous. Of these SCAs, polyglutamine (polyQ) SCAs (SCA1, SCA2, SCA3, SCA6, SCA7, and SCA17) have emerged as the most common subtype (Paulson et al., 2017). Their disease origins, identical to other polyQ diseases [Huntington’s disease (HD), dentatorubral pallidoluysian atrophy (DRPLA), spinal and bulbar muscular atrophy, and X-linked 1 disease (SMAX1/SBMA)], have been traced to the abnormal expansion of CAG repeats in the coding region of disease-associated genes. A brief description of each polyQ SCA is presented in our table below (Table 1).

**TABLE 1:** A list of polyQ SCAs with a brief description of each disease.

PolyQ SCA disease	Proteins	CAG repeats (normal)	CAG repeats (disease)	Normal subcellular localization	Putative NLS/NES	A brief description of the characteristic features of proteotoxicity in each disease
SCA1	Ataxin1	6–39	>39	Predominantly Nucleus, but both Nucleus and Cytoplasm in Purkinje cells	**1 NLS** (Klement et al., 1998)	Neurotoxicity of polyQ SCA1 is associated with its nuclear accumulation, which is affected by the phosphorylation of serine 776 near the NLS.
SCA2	Ataxin2	14–32	34–77	Cytoplasm	–	Intra-nuclear inclusion is considered absent in polyQ SCA2.
SCA3	Ataxin3	12–40	62–86	Predominantly Cytoplasm	**1 NLS** (Tait et al., 1998) **2 NES** (Antony et al., 2009)	Also known as Machado-Joseph disease (MJD), polyQ SCA3 is critically associated with nuclear proteotoxicity following the expansion of polyQ repeats.
SCA6	CACNA1A	4–18	19–33	Membrane associated	–	The accumulation of polyQ SCA6 in nuclear inclusions are associated with cell death. Only cleaved fragments translocate into the nucleus.
SCA7	Ataxin7	4–35	37–306	Nucleus and Cytoplasm	**1 NLS** (Kaytor et al., 1999) **1 NES** (Taylor et al., 2006)	PolyQ SCA7 is critically associated with nuclear proteotoxicity following the expansion of polyQ repeats.
SCA17	TBP	25–43	43–66	Nucleus	–	PolyQ expansion in polyQ SCA17 reduces its association with DNA and induces DNA binding-independent neurotoxicity in the nucleus.

Discrepancies in the affected neural regions, disease severity, and disease phenotype between the polyQ SCAs are well described in a previous review (Buijsen et al., 2019). What is often observed within the subtype (with the exception of polyQ SCA2), however, is the fact that toxic polyQ proteins generated from abnormal CAG repeat expansion lead to their nuclear accumulation in the neuron. In a subset of polyQ SCA diseases (SCA3, SCA6, and SCA7), polyQ-expanded SCA proteins abnormally translocate from their original locations, such as the cytoplasm or cytoplasmic membrane, into the nucleus upon polyQ SCA-associated genetic mutations (Havel et al., 2009). Strikingly, these nuclear polyQ SCA proteins sequester other nuclear proteins, primarily including transcription factors (TFs), thereby disrupting gene transcription. This disruption in gene transcription causes perturbation of the intracellular homeostasis and eventually leads to a variety of neuropathic phenotypes and neuronal cell death.

Despite tremendous amounts of research that have directly linked the cause of polyQ SCAs to a single expansion pattern of genetic aberration, scientists have yet to produce drugs or therapeutic treatments that robustly ameliorate the deteriorating effects of these diseases. The current lack of effective treatment may be attributed to the historical approach of mitigating polyQ SCAs either by reducing the expression (or correcting the misfolding) of polyQ proteins or by enhancing their degradation. The theory behind such approaches is that a reduction in accumulated polyQ proteins lessens their proteotoxicity and provides therapeutic benefit to patients. Though this idea has been widely explored, it remains unclear whether strategies involving the reduction of toxic polyQ proteins are therapeutically feasible given the current technical limitations in gene therapy and in selective clearance of nuclear accumulated toxic polyQ proteins. Researchers now face the challenge of searching for an alternative curative approach to suppress the disease-causing features of nuclear-accumulated polyQ SCA proteins. Thus, it is essential to address fundamental questions, including how CAG repeat expansion takes place, how expanded polyQ SCA proteins accumulate in the nucleus, and how nuclear polyQ SCA proteins form insoluble aggregates that escape degradation. In this review, we summarize the currently available answers to these questions and provide our perspectives on the key issues surrounding polyQ SCAs to better handle the challenges that these diseases present.

## Repeat Instability-Induced Abnormal Expansion of polyQ Repeats in polyQ SCAs

As mentioned above, polyQ SCAs are caused by the expansion of CAG trinucleotide repeats (TNRs) within the coding sequences of disease-associated genomic loci. The abnormal genetic expansion of repeated sequences as units is known as repeat instability, a unique form of mutation that is associated with more than 40 neurological, neuromuscular, and neurodegenerative diseases (Paulson, 2018). Although TNRs (which include CTG, CGG, GAA, and CAG triplets) emerge as the most common repeat-associated unit, other forms of repeat instability mutations, such as pentanucleotides (ATTCT repeat expansion, SCA10) or hexanucleotides (G_4_C_2_ repeat expansion, amyotrophic lateral sclerosis), also exist (Everett and Wood, 2004; Paulson, 2018). An important question worth addressing is how this repeat instability occurs. Although a clear answer remains elusive, accumulating evidence surrounding these questions helps us understand the exact nature of repeat instability-induced expansion of polyQ SCAs.

### Structural Properties of DNA Associated With TNR Expansion

The mechanism of repeat expansion can be potentially inferred by the structural properties of DNA following expansion. Expanded TNRs are intrinsically able to form non-B DNA structural elements (Wells et al., 2005), which may abnormally affect various nucleic acid-mediated cellular processes (including DNA replication and repair, transcription, and chromatin remodeling) and lead to repeat instability of expanded or contracted TNRs (Usdin et al., 2015). For example, the hairpin structure, formed by CAG repeats, is known as an inefficient substrate for replicating DNA polymerases (Liu and Leffak, 2012) and may contribute to the escape from DNA repair in yeast (Moore et al., 1999). Accordingly, strand slippage and hairpin formation within CAG repeats during DNA replication are proposed to be associated with TNR expansion (Iyer et al., 2015). Also, research points out that a stable RNA-DNA hybrid of the CUG-CAG repeat in the R-loop stimulates genetic instability (Lin et al., 2010) and that altered DNA methylation at CpG sites (caused by deficiency of DNMT1) promotes CAG repeat expansion (Dion et al., 2008).

### Genetic Risk Factors Underlying TNR Expansion Identified From Human Patients

The mechanism of TNR expansion may be inferred from the genetic risk factors with which it is associated. In a search for these factors, analysis of single nucleotide polymorphisms (SNPs) in SCA patients showed a significant association between DNA repair genes, including FAN1, PMS2, and RRM2B, and the onset of SCAs (Bettencourt et al., 2016). In addition, a genome-wide association study revealed that genes associated with mismatch repair, including MSH3, MLH1, and PMS1, modify age of onset for HD (Genetic Modifiers of Huntington’s Disease Consortium, 2019). These findings suggest that common DNA repair pathways influence TNR expansion (Jones et al., 2017; Massey and Jones, 2018; Yau et al., 2018).

### Genetic Risk Factors Underlying TNR Expansion Identified From Animal Models

Alternatively, genetic studies in model systems may also identify additional genetic factors associated with TNR expansion. For example, genetic analysis of *in vivo* polyQ SCA models shows the association between repeat instability, transcription-coupled nucleotide excision repair (TC-NER), and regulators of DNA repair. Interestingly, TC-NER, as opposed to mismatch repair, is shown to promote TNR expansion; the knockout of XPA, a component of TC-NER, in neuronal tissues of SCA1 model mice reduces CAG TNRs (Hubert et al., 2011). Thus, given that TC-NER and mismatch repair pathways are regulated through separate routes (Hakem, 2008; Massey and Jones, 2018), the opposing functional roles of DNA repair pathways in TNR expansion implicate a potential presence of pathway-specific perturbation in disease conditions.

In addition, adenosine 3′,5′-monophosphate response element-binding protein (CREB) modulates CAG repeat instability in a *Drosophila* SCA3 model (Jung and Bonini, 2007). Furthermore, the interaction between essential DNA strand break repair enzyme PNKP with mutant polyQ SCA3 leads to persistent accumulation of DNA damage and chronic activation of DNA damage response ATM signaling pathway (Gao et al., 2015); this supports a direct link between mutant polyQ SCAs (at least in polyQ SCA3) and DNA damage.

As mentioned earlier, the repeat instability is associated with more than 40 diseases, some of which likely arise from common genetic or molecular pathways. It is, therefore, reasonable to hypothesize that therapeutic measures that intervene the aberrant repeat expansion of disease-associated genes induced by repeat instability may have broad application across many of the abovementioned diseases, quite possibly beyond polyQ SCAs.

## Nuclear Accumulation of Expanded polyQ SCA Proteins and Their Interactions With Nuclear Proteins

Most proteins carry out specific functions at defined intracellular locations, signifying that an accurate knowledge of protein localization to and within subcellular compartments is essential to understand the function of proteins. In particular, the nuclear import of proteins affects several key cellular processes, including regulation of gene expression, signal transduction, and cell cycle (Marfori et al., 2011). This indicates that nuclear/cytoplasmic transport of proteins is tightly controlled in properly functioning cells.

Nuclear transport occurs through the nuclear pore complexes (NPCs), which are comprised of combinations of approximately 30 different nucleoporins (NUPs) (Beck and Hurt, 2017). For protein cargo bearing classical nuclear localization signal (NLS) sequences, nuclear import is mediated by the formation of an import complex between the cargo and importin-α in the cytoplasm. Subsequently, importin-β recognizes the complex and transports the importin-α-bound cargo across the NPC in a process that involves the FG-nucleoporin in NPCs (Lott and Cingolani, 2011). Proteins smaller than 40 kDa may passively diffuse through NPCs (Dingwall and Laskey, 1986).

An abnormal accumulation of mutated proteins in the nucleus is consistently observed in most polyQ SCAs (with the exception of SCA2). In SCA3, for example, SCA3 proteins originally located at the cytoplasm are abnormally translocated into the neuronal nucleus upon the abnormal expansion of polyQ domain near the NLS (Bichelmeier et al., 2007). Highlighting the importance of nuclear translocation of disease-associated proteins in polyQ SCAs, reports indicate that the reduction of polyQ expanded SCA3 and SCA6 proteins in the nucleus through downregulation of protein import machinery (karyopherin α3) alleviates the degenerating phenotype induced by expanded SCA proteins in *Drosophila* and mouse models (Tsou et al., 2015; Sowa et al., 2018). Although the mechanism by which the preferential nuclear localization of the toxic proteins occurs is not known, posttranslational modification (including phosphorylation), and/or interaction between NUPs and disease-associated expanded polyQ may prompt the NLS-mediated nuclear translocation of a subset of polyQ-expanded proteins (Mueller et al., 2009; Weber et al., 2014).

### Potential Modes of Action (1): Increased Nuclear Retention of polyQ SCAs

**FIGURE 1:**
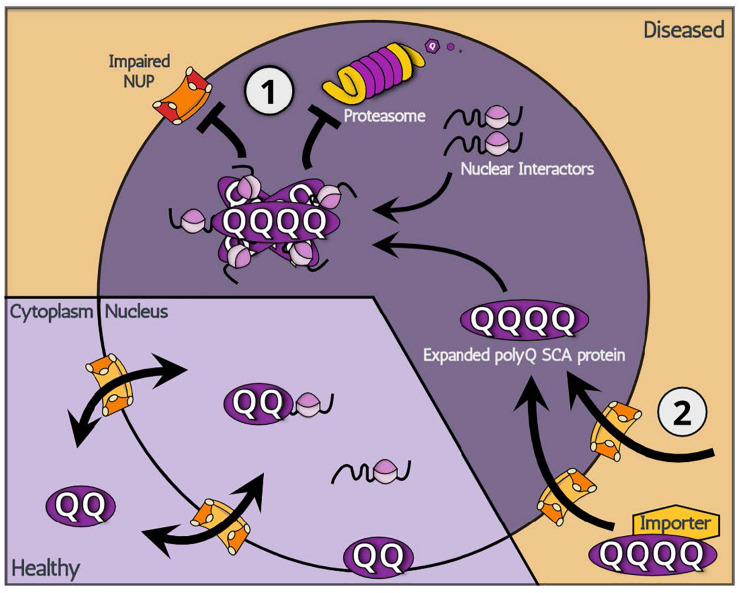
A schematic illustration showing two potential modes of action: (1) increased nuclear retention and (2) increased nuclear import, leading to the nuclear accumulation of polyQ SCA-associated toxic proteins.

In cases of polyQ SCA proteins that originally reside in the nucleus, the abnormal accumulation of mutated proteins is likely related to the inadequate clearance of multimerized toxic proteins (Figure 1). Multimerized polyQ proteins are degraded through limited pathways once inside the nucleus. Autophagy, which is responsible for degradation of comparably large-sized proteins in the cytoplasm, is not available in the nucleus. This leaves the ubiquitin-proteasome system (UPS) as the primary source of protein clearance for multimerized polyQ SCA proteins in the nucleus. In fact, E3 ubiquitin (ub) ligases, proteins responsible for recognizing and recruiting target proteins for ubiquitination, have gained steady attention in polyQ disease research as the E3 ub ligases are reported to have modulating effects on polyQ toxicity regardless of the subcellular location of E3 ub ligase actions by promoting ubiquitination and degradation of polyQ proteins in both cultured cells and transgenic animal models (Bhat et al., 2014; Chhangani et al., 2014). A recent study, for example, identified *F-box* gene *FipoQ* as a novel modifier of polyQ toxicity, whereby the protein interacts with Cul1, a scaffolding protein associated with the Cullin-RING ub ligase complexes, and expanded polyQ proteins to ubiquitinate and degrade the latter via the UPS pathway (Chen et al., 2019). Additionally, evidence indicates that, in SCA7, SUMOylation, a reversible modification that attaches a small ubiquitin-related SUMO protein, recruits RNF4 ub ligase to tag mutated polyQ SCA7 proteins for UPS-mediated degradation (Marinello et al., 2019). Even attempts at engineering promyelocytic leukemia protein, an E3 ub ligase enzyme, to reduce the levels of either the soluble misfolded form of expanded SCA1 or its precipitated aggregate have produced promising results for potential treatments of not only polyQ SCA1, but also other polyQ disorders (Dhar et al., 2020).

Although the UPS degrades monomerized protein groups relatively well, a certain proportion of polyQ SCA proteins exist in the nucleus in a multimerized form, which may be degraded by the UPS in a slow or otherwise incomplete manner. Because the multimerized proteins originate from monomeric polyQ proteins in the cytoplasm, both the UPS and autophagic processes may act to decrease the quantity of nuclear multimerized forms of polyQ proteins by directly acting either on cytoplasmic or on nucleic-residing monomeric polyQ proteins. Thus, both autophagic processes and the UPS have the potential to ameliorate toxic effects of expanded polyQ proteins. For example, a study identified autophagy-promoting compounds that reduce the levels of expanded polyglutamine proteins in cultured cells (Zhang et al., 2007). Furthermore, treatment of polyQ SCA3 patient-derived iPSC lines with rapamycin, an autophagy inducer, resulted in increased degradation of expanded SCA3 proteins (Ou et al., 2016). However, as autophagy can only eliminate cytoplasmic mutant proteins, the autophagic clearance of expanded polyQ proteins may be best suited for SCA2 where toxic proteins accumulate in the cytoplasm.

The introduction of mutated polyQ SCA may also directly disturb the UPS. Reports indicate that the overexpression of polyQ SCA3 perturbs the function of the UPS, ultimately leading to the abnormal increase in proteasome substrates (Burnett et al., 2003). Expanded polyQ SCA may also indirectly cause nuclear UPS malfunction by sequestering in the cytoplasm key chaperones that translocate cargo molecules to the nuclear UPS (Park et al., 2013). Although it remains unclear whether this impairment occurs in the cytoplasm or the nucleus, the data suggest that the expanded polyQ proteins may overwhelm the UPS and jeopardize cellular homeostasis. Likewise, if the abnormal expansion of polyQ proteins stimulate multimerization and ultimately block proper UPS-mediated clearance of monomeric polyQ proteins as was shown for polyQ huntingtin in the cytoplasm (Schipper-Krom et al., 2012), the nuclear accumulation of polyQ proteins may clog the proteasome and lead to a vicious cycle that results in an increase in polyQ proteins as well as other nuclear protein residues. In addition, a recent report showed an interesting association between SCA3 function and autophagosome formation through its regulatory effects on the level of beclin1 (Ashkenazi et al., 2017), a key initiator of autophagy (Liang et al., 1999). The expansion of polyQ in SCA3 decreased its deubiquitinase activity toward beclin1 and resulted in the decrease of beclin1 levels and impairment of starvation-induced autophagy (Ashkenazi et al., 2017). These impairments in UPS and autophagy induced by polyQ SCAs may further increase the amount of expanded polyQ SCAs and associated neuronal toxicity.

### Potential Modes of Action (2): Increased Nuclear Import of polyQ SCAs

For SCA3, SCA6 (cleaved protein only), and SCA7, where the expanded polyQ SCA proteins abnormally translocate into the nucleus, there are few known determinants that cause polyQ SCA proteins to translocate into the nucleus. However, several disease-associated features are suspected to be contributing factors. The first is, as can be expected, the expansion of Q-repeat itself. However, the fact that the translocation to the nucleus is not observed in SCA2 even with polyQ expansion (see below for discussion) implies that the expansion of CAG repeat is not sufficient for nuclear translocation. The second factor is the cleavage of proteins. It is known that polyQ proteins undergo proteolytic cleavage by specific proteases and that cleaved proteins are more toxic and more likely to form nuclear aggregates than full-length ones (Friedman et al., 2008). This second factor may be associated with the protein size threshold for free trafficking across nuclear pores: polyQ SCA proteins that are small enough to freely diffuse across the nuclear envelope may accumulate in the nucleus. Other factors, such as molecular inducers, are not well characterized at this time, however, it is important to point out that proteases associated with the direct regulation of polyQ SCA3 cleavage have been reported to be therapeutic in fly models (Jung et al., 2009).

Regarding the mechanism underpinning nuclear translocation of polyQ SCAs, two major hypotheses can be made (Figure 1). The first relates to the aberrant gain of function of new import properties linked with expanded polyQ regions: the abnormal expansion of polyQ proteins expose a hidden NLS domain within their structure, which signals for nuclear import. It is also possible that an unknown factor recognizes Q-expanded domains and imports polyQ SCA proteins. However, the existence of an unknown factor seems unlikely given that polyQ SCA2 remains in the cytoplasm yet contains an expanded polyQ domain that exerts the disease-causing toxicity. Evidence for the aberrant gain of function is indeed scarce, leaving the following hypothesis more likely. Although they contain an NLS domain, polyQ SCA proteins SCA3 and SCA7 appear to initially localize primarily in the cytoplasm. From this observation, it is conceivable that these polyQ SCA proteins, in fact, shuttle between the nucleus and cytoplasm though, for unknown reasons, primarily locate in the cytoplasm. In other words, it is possible that, while shuttling, expanded polyQ SCA proteins become unable to exit the nucleus, effectively being trapped inside the nucleus. Consistent with this idea, previous reports indicate that nuclear polyQ proteins directly impair nuclear pores and/or machineries involved in protein nuclear cytoplasmic transport (Cornett et al., 2005). Also, the nucleus already has many potential Q-rich binding partners that can readily interact with polyQ proteins. In addition, the fragmented SCA6 proteins may be sufficiently small to translocate into the nucleus via passive diffusion and then become trapped in a similar manner to SCA3 and SCA7. The nuclear accumulation of expanded polyQ SCA proteins may be caused by abnormal entanglement with nuclear interacting proteins.

### Abnormal Interactions of Nuclear polyQ SCAs With Essential Nuclear Proteins

Once expanded polyQ proteins accumulate in the nucleus, they sequester various nuclear proteins and alter RNA homeostasis (Chung et al., 2018). Interestingly, certain TFs containing Q-rich domains in the non-pathological range, such as CREB-binding protein (CBP) and TATA-binding protein (TBP) (Sakahira et al., 2002), are frequently detected in the neuronal inclusions, indicating that nuclear polyQ SCA proteins sequester these often-essential nuclear proteins via polyQ-to-polyQ or polyQ-to-Q-rich domain interactions, a previously established coiled-coil–mediated mode of protein-to-protein interactions (Schaefer et al., 2012). Though the exact structural properties of polyQ multimers remain undetermined due to the technical limitations in obtaining soluble samples required for X-ray crystallography or conventional NMR studies, an accumulated body of literature indicates that polyQ multimers form long stretches of self-associating β-sheets (Bevivino and Loll, 2001; Tanaka et al., 2001; Vitalis et al., 2009). Additionally, recent work in fruit flies has demonstrated that polyQ multimers can also form coiled-coil super secondary structures. This coiled-coil structure was determined to play a major role in sequestering other coiled-coil–bearing nuclear proteins, such as FOXO (Kwon et al., 2018), suggesting that TFs are hijacked by coiled-coil–bearing polyQ tracks. Although coiled-coil–forming proteins normally have specificity in their structural patterns, such as dimers, trimers, and tetramers (Grigoryan and Keating, 2008), it remains unclear whether polyQ proteins form specific patterns of coiled-coil structure with their interacting partners. Further studies are required to understand the nature of coiled-coil–mediated interactions between polyQ SCAs and their targets.

Aggregation itself, however, may not be the main contributing factor to polyQ toxicity. There are ongoing debates in the field of polyQ disease as to whether or not insoluble aggregates are, in fact, more toxic than soluble oligomers. In the case of Huntington’s disease, soluble oligomers of polyQ-expanded huntingtin engage more extensively with key proteins (Kim et al., 2016). This implies that the aggregation of polyQ proteins into an insoluble form is a cellular defense mechanism employed to minimize the spread of the more damaging toxic effect of soluble polyQ monomers/oligomers within the cell. Regardless of its aggregation status, however, the presence of polyQ protein in the nucleus alters RNA homeostasis by abnormally interacting with nuclear proteins via polyQ-to-polyQ, coiled-coil-to-coiled-coil, and non-specific modes of interactions that lead to the trapping of TFs. Although it remains unclear how the structural properties of polyQ proteins translate into the often-deleterious toxicity, the abnormal interactions with essential nuclear proteins likely play a key role in the pathological mechanism that disturbs the state of the cell.

## Discussion

So far, we have reviewed current updates on the cell biological mechanisms underpinning the polyQ expansion and nuclear accumulation of disease-associated polyQ SCA proteins. However, these mark only the beginning stages of the polyQ SCA development as the accumulation of polyQ proteins in the nucleus subsequently leads to a multitude of cellular impairments, which ultimately cause neuronal cell death characteristic of polyQ SCAs. The nature of broad interactions of toxic proteins with various TFs implies a wide range of deleterious cellular defects, including dendrite defects (Kweon et al., 2017), caused by expanded polyQ proteins. These aberrations are observed in the cerebellum, brainstem, and spinal tracts of polyQ SCA patients (Buijsen et al., 2019), most of which are associated with nuclear accumulated polyQ proteins (with the exception of polyQ SCA2). Dendrites, for example, form distinct pathogenic patterns caused by specific alteration in the dendritic F-actin structure and accompanied by reduced RNA granule formation when nuclear polyQ SCA protein accumulation is induced (Lee et al., 2011). Nuclear polyQ accumulation also perturbs satellite organelles, such as Golgi outposts, that support the dendrites by supplying local plasma membrane (Chung et al., 2017). Neurons function with the support of myriad complicated processes, the majority of which rely on appropriate RNA homeostasis. Prolonged defects in RNA regulation lead to malfunctioning neurons, which may characterize altered animal behavior and cell death.

Due to the practical limitations surrounding the characterization of pathogenic mechanisms of polyQ SCAs in human patients, animal models overexpressing expanded polyQ proteins are often used. These animal models, including fruit fly (Long et al., 2014; Xu et al., 2015), mouse (Figiel et al., 2012), and non-human primates (Tomioka et al., 2017), show brain atrophy similar to that of human polyQ SCA patients. PolyQ diseases are particularly amenable to modeling in a variety of animals because the triggering event (i.e., repeat expansion and accumulation of toxic polyQ proteins) is so clearly defined. A striking feature of the polyQ disease model is that many mouse models for a subset of polyQ SCAs that are accompanied by nuclear proteotoxicity share common phenotypes. This phenomenon resembles the situation in human patients in which many, but not all, SCA disorders are clinically indistinguishable based on neurological examination alone (Figiel et al., 2012). This observation led to the hypothesis that all polyQ disorders accompanied by nuclear proteotoxicity are, in fact, one disease that may have a common treatment. However, this hopeful speculation is complicated by human case reports indicating the existence of disease subtype-specific features of polyQ SCAs. The specific characteristics associated with each subtype of polyQ SCA is documented in both a previous report (Buijsen et al., 2019) and our table (Table 1).

Here, we present a model in which nuclear accumulation of polyQ SCA proteins may explain the subtype-specific characteristics of the diseases. Based on the fact that CAG expansion occurs in subtype-specific genetic loci for each subtype of polyQ SCA, our model involves each subtype of expanded polyQ proteins having an affinity toward subtype-specific nuclear proteins. Although all polyQ proteins have an expanded CAG domain, regions that surround the polyQ repeat regions contain subtype-specific flanking residues. These residues may also exert toxicity by abnormally trapping their interacting partners during multimerization of the mutated proteins. Therefore, the differences in protein residues that flank the polyQ expanded region might give rise to subtype-specific interactions with nuclear proteins. More research elucidating the interacting partners of nuclear polyQ proteins is needed to better understand polyQ SCA diseases.

Although there are currently no known cures for polyQ SCAs, there are some promising avenues worth exploring. Therapies that target structural properties of polyQ SCAs may be useful in ameliorating toxicity of disease-associated proteins. For example, QBP1 is a peptide with selective affinity toward an expanded form of polyQ repeats containing β-sheet structures and acts as a structural inhibitor of polyQ protein multimerization (Nagai et al., 2000). Interventions, such as QBP1, which target specific structures associated with multimerization of disease-related proteins are steadily gaining attention for their effectiveness as therapeutic agents for neurodegenerative diseases, including Alzheimer’s disease (Sievers et al., 2011). Although this strategy of direct inhibition of toxic structural properties of polyQ SCAs shows obvious therapeutic effects in model organisms (mouse, Popiel et al., 2009; fruit fly, Nagai et al., 2003), we believe the development of drugs that specifically lower nuclear toxicity of polyQ SCA proteins either through blocking nuclear translocation or by inhibiting repeat instability would greatly complement the therapeutic strategy described above. It is also worth pointing out that if, as we mentioned previously, promoting the aggregation of polyQ proteins is, in fact, protective for neurons, therapeutics to enhance the aggregation of polyQ proteins and reduce the net toxicity of polyQ proteins would also be potentially beneficial.

## Author Contributions

DL, Y-IL, Y-SL, and SL conceptualized the theme of the review and wrote the manuscript together.

## Conflict of Interest

The authors declare that the research was conducted in the absence of any commercial or financial relationships that could be construed as a potential conflict of interest.
